# Decentralised ECG monitoring for drug-resistant TB patients in ambulatory settings

**DOI:** 10.5588/ijtldopen.23.0623

**Published:** 2024-04-01

**Authors:** M. Mbenga, A. Slyzkyi, V. Mirtskhulava, S. Pak, A. Gebhard, G. Utepkalieva, A. Sagimbekova, M. Adenov, G. Ryskulov

**Affiliations:** ^1^ KNCV Tuberculosis Foundation, The Hague, The Netherlands;; ^2^ KNCV Kazakhstan, Almaty,; ^3^ National Scientific Centre of Phthisiopulmonology, Almaty, Republic of Kazakhstan.

**Keywords:** drug-resistant TB, ECG device, safety monitoring

Dear Editor,

Managing drug-resistant TB (DR-TB) poses substantial challenges to patients and health systems globally.^1^ The emergence of new TB drugs such as bedaquiline, pretomanid and delamanid, and the repurposing of fluoroquinolones, clofazimine and linezolid, has marked significant progress in developing shorter, safer and more tolerable regimens for DR-TB.^2^ However, it is important to acknowledge that the use of bedaquiline, delamanid, fluoroquinolones and clofazimine come with the potential risk of fatal arrhythmias associated with QT prolongation.^3–7^ To address this concern, electrocardiogram (ECG) monitoring is crucial throughout treatment, which is integrated into active drug safety monitoring and management (aDSM).^4–8^ Unfortunately, access to ECG is often centralised, requiring patients to travel long distances, thereby introducing practical and logistical challenges. Kazakhstan, a high-burden TB country,^9^ has introduced new treatment regimens to enhance the management of DR-TB.^9^ The delivery of TB services is distributed across TB dispensaries, primary healthcare facilities and TB hospitals. To note, DR-TB treatment is initiated in TB hospitals, after which treatment monitoring (including ECG) continues on an ambulatory basis.^10,11^ In this context, the KardiaMobile 6L (KM6L; Alivecor, Mountain View, California, USA), which serves as a point-of-care tool for ECG, may have specific advantages for populations facing challenges in accessing healthcare facilities. The KM6L device is equipped with three electrodes for recording a clinical-grade 6-lead ECG and transmits data wirelessly to a smartphone or tablet. Food & Drug Administration clearance validates its ability to detect atrial fibrillation, bradycardia, tachycardia, sinus rhythm with premature ventricular contractions, sinus rhythm with wide QRS, and sinus rhythm with supraventricular ectopy.^12–14^ Additionally, it has been approved for monitoring QT prolongation, making it relevant for patients undergoing TB treatment with potentially cardiotoxic drugs.^15^ To assess the feasibility and acceptability of integrating KM6L into ambulatory settings for QT prolongation monitoring, we conducted a study involving adult DR-TB patients receiving QT-prolonging drugs.

The study involved a questionnaire-based survey administered before and after the intervention with KM6L. Patients undergoing DR-TB treatment with QT-prolonging drugs were screened by doctors at the National Scientific Centre of Phthisiopulmonology, Almaty, Republic of Kazakhstan, using inclusion criteria (above 18 years). Those meeting the eligibility criteria were provided with study information and signed informed consent forms. The study was approved by Kazakh National Tuberculosis Programme (NTP). Participants underwent training on the use of KM6L and software. Initial ECG recordings were performed under the supervision of healthcare workers (HCWs). Each participant received a KM6L and a smartphone or tablet with internet access for the study duration, ranging from 3 to 6 months. The study aimed to enrol 100 participants, each of whom, along with the HCWs, completed a baseline questionnaire before using KM6L, as well as a post-test questionnaire. Four data collection instruments were utilised: a screening form, a baseline questionnaire, an endline questionnaire and a HCW questionnaire. All forms were administered in the Russian language using digital Google Form (Google, Mountain View, CA, USA) formats. The screening form captured demographics, details of the TB/DR-TB treatment administered (specifically QT-prolonging drugs), screening for TB symptoms, blood electrolyte levels (K+ and Mg++), ECG (QTcF grade) and concomitant medications. This form was completed during the screening stage to assess patient eligibility. On enrolment, HCWs administered the baseline questionnaire, focused on patients' experiences with conventional 12-lead ECG. The questionnaire captured time taken for the ECG procedure, associated expenses, the need to travel to a HCF solely for ECG, the patient's comfort and pain levels, irritability and anxiety. After use of KM6L to record ECG, participants completed the endline questionnaire, capturing the location of the ECG procedure (home vs. healthcare facility), assessment of the ease, comfort and expectations associated with KM6L use, acceptability of using KM6L at home and challenges encountered during ECG recording and data transfer. HCWs experiences of KM6L were captured through a self-administered questionnaire during home visits, which registered occupation, place of work, the number of ECGs performed daily and the time spent on conventional and KM6L ECG recording. Also, HCWs rated KM6L on a 5-point Likert scale for ease of use, comfort and expectations in ambulatory settings. The responses in CSV format were analysed using Stata v17.0 (Stata, College Station, TX, USA). Descriptive analysis included frequency calculations for qualitative data and medians with interquartile ranges for continuous quantitative data. The 5-point Likert scale questions were analysed using mean and standard deviation.

From a total of 124 candidates, 81 accepted and provided written consent; 64.2% of the participants were male, with a median age of 36 years (interquartile range [IQR] 26–47). All 81 participants completed the baseline questionnaire, and 69 completed the endline questionnaire after using KM6L (Table Part A). To note, 79% of patients used the KM6L at home, spending a median time of 4 min on the ECG procedure. We observed 100% compliance with the scheduled ECG monitoring in ambulatory settings, indicating a high level of acceptance and adherence. Both patients and HCWs reported positive impacts associated with the use of KM6L. Patients appreciated the reduced time and travel expenses as they no longer needed to travel to and from healthcare facilities solely for ECG (Table Part B). This made the monitoring process more streamlined but also positively influenced patient comfort and overall satisfaction. However, the ECG data were not used for clinical management, but as an indication as to whether to invite a patient to the healthcare facility for further assessment. This innovative approach of monitoring QT prolongation promotes a people-centred approach and addresses logistical challenges associated with traditional ECG. The technology facilitates ECG recordings as frequently as necessary, potentially allowing for the early detection of cardiotoxicity. Affordability and minimal running costs enhance the attractiveness of KM6L for remote monitoring. Additionally, the study revealed that individuals with no experience in using smart devices were able to independently use innovative technology to routinely monitor their own safety from the comfort of their homes. This finding underscores the feasibility and acceptability of incorporating digital health technologies into DR-TB care.

**Table.** Participant characteristics and experience of using ECG procedures. **A)** Participant characteristics (*n* = 81).

Characteristic	*n* (%)
Age, years, median [IQR]	36 [26–47]
Age group, years	
<24	13 (16.1)
25–34	26 (32.1)
35–44	13 (16.1)
45–54	18 (22.2)
>55	11 (13.6)
Male sex	52 (64.2)
Previous DS-TB treatment history	12 (14.8)
Previous DR-TB treatment history	8 (9.9)

**Table:** **B)** Patients with experience with ECG procedure prior to start using KM6L: baseline characteristics (*n* = 81).

Characteristic	*n* (%)
Time spent on the ECG procedure at the HCFs, min, median [IQR]	10 [10–15]
Time spent on travel to and from the HCF, min, median [IQR]	40 [30–60]
Total time spent on the ECG procedure in the HCF, including travel and waiting time, min, median [IQR]	90 [60–120]
Expenses for travel to and from HCF, USD, median [IQR]	1.15 [0.37–3.45]
Those who experienced expenses related to travel to and from the HCF	56 (69.1)
Those who visited HCF for ECG procedure only	14 (17.3)
Frequency of ECG procedure in HCF	
At least once a month	29 (35.8)
Less than once per month	44 (54.3)
Unknown	8 (9.9)
Those who considered ECG procedure in HCF comfortable	74 (91.6)
Experience with ECG using KM6L (the 5-point Likert scale questions) (*n* = 69), mean ± SD	
Considered KM6L easy to use from easy (1) to difficult (5)	1.65 ± 1.19
Convenience of using the KM6L device compared to a conventional ECG from uncomfortable (1) to comfortable (5)	4.49 ± 0.98
The device met expectations compared to a conventional ECG from no (1) to yes (5)	4.47 ± 0.99
Overall KM6L satisfaction from low (1) to high (5)	4.52 ± 0.88

IQR = interquartile range; DS-TB = drug-susceptible TB; DR-TB = drug-resistant TB; ECG = electrocardiogram; HCF = healthcare facility; SD = standard deviation; USD = US dollar.

The results of this study contribute valuable insights to the global knowledge and experience of using digital technologies in DR-TB care. The positive outcomes support the potential rollout of KM6L in Kazakhstan and beyond, offering a promising avenue for improving patient-centred care. However, certain limitations should also be considered. KM6L was not registered in Kazakhstan, posing regulatory challenges for its implementation. The small sample size may limit the ability to identify statistically significant associations between issues related to KM6L usage and its combination with other technologies. The study did not explore the impact of patients coming to the healthcare facility for other investigations. A combined study is recommended to assess its overall impact on improving care in ambulatory settings.

In conclusion, our study demonstrates the feasibility and acceptability of integrating KM6L into ambulatory settings for monitoring QT prolongation in DR-TB patients. The positive feedback from patients and HCWs, coupled with the observed benefits in terms of reduced time and travel expenses, supports the potential incorporation of KM6L into DR-TB care in Kazakhstan and other settings.
